# Is intravenous vitamin C contraindicated in patients with G6PD deficiency?

**DOI:** 10.1186/s13054-019-2397-6

**Published:** 2019-04-03

**Authors:** Paul E. Marik

**Affiliations:** Division of Pulmonary and Critical Care Medicine, Eastern Virginia Medical School, 825 Fairfax Av, Suite 410, Norfolk, VA 23507 USA

**Keywords:** Vitamin C, G6PD deficiency, Sepsis, Septic shock, Clinical trials

There is increasing interest in the use of intravenous vitamin C as adjunctive treatment in the management of patients with sepsis and septic shock. Currently, there are at least 20 randomized controlled trials worldwide testing this intervention in patients with sepsis. Almost all of these trials list glucose-6-phosphate dehydrogenase (G6PD) deficiency as an exclusion criterion. This is based on a handful of cases of hemolysis in patients with G6PD deficiency who received large pharmacologic doses of IV vitamin C (> 60 g) [1]. However, the reality is that low-moderate dose intravenous vitamin C may be the treatment of choice for drug-induced hemolysis in patients with G6PD deficiency. In vitro data dating back to 1979 has demonstrated that vitamin C in plasma concentrations up to 5 mmol/l inhibited the oxidation of oxyhemoglobin and Heinz body formation in G6PD-deficient red cells incubated with acetylphenylhydrazine (a strong oxidizing drug) [2]. Serum concentrations of vitamin C are typically in the range of 200–600 umol/l when dosed with 1.5 g IV q 6 hourly. Furthermore, case reports and case series have demonstrated a dramatic reduction of methemoglobinemia and hemolysis in patients treated with intravenous vitamin C in a dose between 1 and 10 g q 6 hourly [3, 4]. Indeed, intravenous vitamin C may be the treatment of choice in G6PD-deficient patients with drug-induced hemolysis, as methyl blue is contraindicated in these patients [3]. These data suggest that in the dosage currently under investigation (6 g/day), vitamin C should not be considered contraindicated in patients with known or suspected G6PD deficiency. This is important, as GDPD deficiency is not uncommon in patients of African and Mediterranean descent [5]. Furthermore, sepsis per se may cause methemoglobinemia in both G6PD-deficient patients and those with normal G6PD function.
